# Deficiency and Also Transgenic Overexpression of *Timp-3* Both Lead to Compromised Bone Mass and Architecture *In Vivo*

**DOI:** 10.1371/journal.pone.0159657

**Published:** 2016-08-12

**Authors:** Behzad Javaheri, Mark Hopkinson, Blandine Poulet, Andrea S. Pollard, Sandra J. Shefelbine, Yu-Mei Chang, Philippa Francis-West, George Bou-Gharios, Andrew A. Pitsillides

**Affiliations:** 1 Department of Comparative Biomedical Sciences, The Royal Veterinary College, London, United Kingdom; 2 Institute of Ageing and Chronic Disease, University of Liverpool, Liverpool, United Kingdom; 3 Department of Mechanical and Industrial Engineering, Northeastern University, Boston, Massachusetts, United States of America; 4 Department of Craniofacial Development and Stem Cell Biology, King's College London, London, United Kingdom; Universite de Lyon, FRANCE

## Abstract

Tissue inhibitor of metalloproteinases-3 (TIMP-3) regulates extracellular matrix via its inhibition of matrix metalloproteinases and membrane-bound sheddases. *Timp-3* is expressed at multiple sites of extensive tissue remodelling. This extends to bone where its role, however, remains largely unresolved. In this study, we have used Micro-CT to assess bone mass and architecture, histological and histochemical evaluation to characterise the skeletal phenotype of *Timp-3* KO mice and have complemented this by also examining similar indices in mice harbouring a *Timp-3* transgene driven via a Col-2a-driven promoter to specifically target overexpression to chondrocytes. Our data show that *Timp-3* deficiency compromises tibial bone mass and structure in both cortical and trabecular compartments, with corresponding increases in osteoclasts. Transgenic overexpression also generates defects in tibial structure predominantly in the cortical bone along the entire shaft without significant increases in osteoclasts. These alterations in cortical mass significantly compromise predicted tibial load-bearing resistance to torsion in both genotypes. Neither *Timp-3* KO nor transgenic mouse growth plates are significantly affected. The impact of *Timp-3* deficiency and of transgenic overexpression extends to produce modification in craniofacial bones of both endochondral and intramembranous origins. These data indicate that the levels of *Timp-3* are crucial in the attainment of functionally-appropriate bone mass and architecture and that this arises from chondrogenic and osteogenic lineages.

## Introduction

Bone comprises a predominantly type I collagen-rich, mineralised extracellular matrix (ECM) that is synthesised by osteoblasts, degraded by osteoclasts and populated by osteocytes. All bones of the appendicular skeleton form via endochondral ossification, involving calcification of a collagen type II-rich ECM followed by its replacement with bone. In contrast, some bones of the cranial skeleton can also form via intramembranous ossification, the direct differentiation of mesenchymal cells into osteoblasts [1]. The fate of mesenchymal cells and directions of this skeletal differentiation are governed mainly by different signalling pathways [2].

The mechanisms controlling appropriate assembly, organisation, composition and regulation of bone ECM during embryonic development, morphogenesis, tissue remodelling and repair remain however incompletely resolved. Many factors including the Metzincin family, of which the matrix metalloproteinases (MMPs) sub-family include collagenases, gelatinases, stromelysins, matrilysins, membrane-type MMPs participate in this ECM regulation [3]. In addition, other enzymes such as “a disintegrin and metalloproteinase with thrombospondin motifs” (ADAMTS) [4] and ADAM, often called “sheddases”, also affect cellular behaviour by proteolytically releasing extracellular domains of cell surface molecules such as membrane-bound growth factors, cytokines and their receptors [5].

MMP and ADAMTS activities are precisely regulated under physiological conditions by endogenous tissue inhibitors of metalloproteinases (TIMPs 1–4). These four TIMPs differ in their affinities, with TIMP-3 displaying unique molecular features and the broadest inhibition [6–12]. Unlike all other soluble TIMP family members [13–15], TIMP-3 becomes tightly bound to ECM via unique basic domains at both C- and N-termini. This unique TIMP-3 ECM-binding facilitates interaction with heparan and chondroitin sulfate and inhibition of MMPs and membrane-bound sheddases. In addition, TIMP-3 can also inhibit membrane bound and transmembrane ADAM-17 and ADAMTS-4/-5 [16–20]. TIMP-3 is expressed broadly at multiple sites of extensive tissue remodelling such as in embryonic somites, lung, skin as well as interdigit webs [21]. In adult mice, TIMP-3 mRNA and protein have been detected in the kidney cortex, liver, spleen, muscle, heart, brain, ovarian follicles, testis and hair follicles [19, 21]. Despite this intimate spatial distribution and function of TIMP-3 in ECM, its roles in regulation and remodelling of bone ECM are incompletely defined.

MMPs and TIMPs are known to play a crucial role in regulating bone mass and structure [22, 23]. Previous studies have reported that MMP-2, MMP-9 and MT1-MMP act as bone-degrading proteases [24–26] and that mice lacking MMP-13 show increased bone volume due to decreased osteoclast function [27, 28]. TIMP-3 is also found to be expressed in adult bones [21] and long-term huTIMP-3 over-expression in murine hematopoietic cells resulted in late onset osteosclerosis and an increase in trabecular bone volume, attributable to elevated bone formation [29]. Leco *et al*., (2001) reported that *Timp-3* KO mice have normal life span with no significant size/weight differences compared with wild-type pups or adults [30].

More recently, Sahebjam *et al*., (2007) reported that *Timp-3* KO mice show delayed secondary ossification centre formation and spontaneous osteoarthritis soon after birth [31], suggesting that *Timp-3* may affect endochondral ossification. This cartilage-to-bone transition involves sequentially proliferation, differentiation and hypertrophy of chondrocytes and ECM calcification. It is generally held that most hypertrophic chondrocytes undergo apoptosis, however, recent studies suggest that at least some survive this transition to differentiate into osteoblasts and thus contribute to long bone formation and maintenance [32–36].

The extent to which TIMP-3 contributes to the regulation of bone mass and architecture *in vivo* remains unresolved and to the best of our knowledge, no previous study including the original work by Lecco *et al*., (2001) examined the effect of *Timp-3* deficiency on bone mass and organisation. Herein, high resolution micro-computed tomography and static histomorphometry are used to address the hypothesis that *Timp-3* deficiency compromises bone mass and architecture in bones derived by both endochondral and intramembranous ossification [37, 38]. In addition, we have explored the extent to which TIMP-3 contributes specifically to endochondral bone formation by analysing bones from a newly generated transgenic gain-of-function mutant in which *Timp-3* overexpression is driven via Col2a1 chondrocyte-specific enhancer. We hypothesized that such cartilage-specific *Timp-3* overexpression would produce an opposing effect, to enhance bone mass and architecture in bones derived by endochondral, but not intramembranous ossification.

## Materials and Methods

### Animal models

Mice (C57BL6 strain) genetically deficient in *Timp-3* were a gift from Dr Rama Khokha [30]. For transgenic mice, a construct containing collagen IIα1 chain (Col-2a1) proximal promoter region (3000 bp), the first exon (237 bp), the first intron (3020 bp) (gift from B. de Crombrugghe; Zhou et al., 1995), was used to drive expression of human TIMP-3, an IRES (internal ribosomal entry site) sequence and LacZ with a nuclear localizing signal. Not I was used to remove the back-bone vector (pBluescript) and to produce an 11.3-kb fragment that was microinjected into fertilized -C57BL/10 × CBA F1 eggs. Founder mice were identified by analysis of genomic DNA. *Timp-3* mRNA expression in E15.5 embryos were confirmed by wholemount ß-galactosidase staining and qRT-PCR with human TIMP-3 specific primer and probe (QuantiProbe® kit, Qiagen). Homozygous and heterozygous transgenic mice were identified with TaqMan probe for beta-galactosidase (sence: 5’- GTG CAC GGC AGA TAC ACT TG-3’, antisence: 5’- AAC GGT AAT CGC CAT TTG ACC AC-3’, TaqMan probe; 5’ FAM-TCA GCC GGA AAA CCT ACC GGA TTG A–BHQ 3’) and mouse 18S (sence: 5’- GAC CAT AAA CGA TGC CGA CTG -3’, antisence: 5’- CCC TTC CGT CAA TTC CTT TAA G -3’, TaqMan probe; 5’ HEX- CTT CCG GGA AAC CAA AGT CT–BHQ 3’) as described previously [39]. Mice were housed in individually ventilated cages with wood chip and paper bedding and provided standard rodent maintenance diet (Special Diet Services, South Witham, UK) and water ad libitum throughout the study and were euthanized by cervical dislocation. All procedures complied with the UK Animals (Scientific Procedures) Act 1986 and were reviewed and approved by the ethics committee of the Royal Veterinary College (London, UK) and University of Liverpool (Liverpool, UK) and comply with the ARRIVE guidelines (although weight was not recorded) [40].

### High-resolution micro-computed tomography (Micro-CT)

Micro-CT scanning and analysis were performed as described previously [41]. Briefly, tibiae and heads from 8 week old (n = 6 for KO and Tg and n = 5 for WT groups) male *Timp-3* KO and their WT littermates (WT_B6_) and *Timp-3* Tg and their corresponding WT littermates (WT_F1_) were scanned using the Skyscan 1172 (Skyscan, Kontich, Belgium), with x-ray tube operated at 50kV and 200 Micro-A, 1600 ms exposure time with a 0.5 mm aluminium filter and a voxel size of 5 (tibiae) and 6 micro-m (head). The scanning time for each sample was approximately 2 and 3 hours respectively. The slices were then reconstructed using NRecon 1.6.9.4 (Skyscan, Kontich, Belgium). 2D/3D analyses were performed using CTAn 1.15.4.0+ version software (Skyscan, Kontich, Belgium). Additionally, 3D visualization and production of colour-coded images of trabecular, cortical and skull bones were conducted using Avizo 9.0.0 software (FEI, Oregon USA). Finally, phantom calibrated Micro-CT was used to assess cortical tissue mineral density (TMD) on a stack of 100 slices for cortical region at 50% of total tibial length using two Skyscan-supplied bone phantoms with known mineral density values of 0.25 and 0.75 g/cm3 calcium hydroxyapatite.

### Morphometrical analysis

1. **Trabecular analysis**. Prior to analysis, Micro-CT images were re-oriented in DataViewer 1.5.0 (Skyscan, Kontich, Belgium), such that the cross-section within the transverse plane was perpendicular to the long axis of the bone. Tibial length was measured in CTAn 1.15.4.0+ software using a straight line measuring tool and the appearance of the trabecular ‘bridge’ connecting the two primary spongiosa bone ‘islands’ was set as reference point for analysis of the metaphyseal trabecular bone adjacent to the epiphyseal growth plate. 5% of the total bone length from this point (towards the diaphysis) was utilised for trabecular analysis of the proximal tibia. The trabecular region of interest was drawn freehand using the selection tool of CTAn, a few voxels away from the endocortical surfaces in order to avoid inclusion of remnants of primary spongiosa associated with cortical bone. The selected trabecular regions of interests were analysed using CTAn BatMan software (Skyscan, Kontich, Belgium) and morphometric parameters were recorded.
2. **Whole bone cortical analysis**. Whole bone analysis was performed on datasets derived from CT scans using BoneJ [42] (version 1.4.0), an ImageJ plugin [43]. Following segmentation, alignment and removal of fibula from the dataset, a minimum bone threshold was selected using a histogram based method in ImageJ which utilises all pixels in a stack to construct a histogram and was further confirmed using ImageJ “threshold function”. The threshold ranged between 22000–22100 and was applied to all datasets to separate higher density bone from soft tissues and air. This threshold was used in “Slice Geometry” function within BoneJ to calculate bone cross sectional area (CSA), second moment of area around the minor axis (I_min_), second moment of area around the major axis (I_max_), mean thickness determined by local thickness in two dimensions (Ct.Th), ellipticity and resistance to torsion (J). The most proximal (15%) and the most distal portions (25%) of tibial length were excluded from analysis, as these regions include trabecular bone
3. **Craniometric measurements**. Skull dimensions were measured using reconstructed projection images, followed by re-orientation in DataViewer 1.5.1.2 (Skyscan, Kontich, Belgium) so that the sagittal suture is parallel to the viewing plane. The volume of tomograms were rendered using CTvox 3.1.0 r1167 (Skyscan, Kontich, Belgium) to obtain 3D models of the skull which were used to measure cranial length, the distance between the internasal (top of the nose) and the occipital point (the most distal point of the occipital bone), inter-nasal distance (measured between both nasal lateral points), inter-orbitary length (measured between right and left infraorbital foramina) and bi-temporal distance measured in the more distant point of the jugal process off squamosal with respect to the sagittal plane using ImageJ. Mandibular measurements were made using 3D generated models by Mimics Research 17 (Materialise, Belgium). All measurements from recognizable geometric morphometric landmarks on digitized images of the skull were performed as described previously [44, 45].

### Histologic analysis

Tibiae from all groups were dissected, fixed for 24 hours in neutral buffered formalin, decalcified in EDTA and processed for standard paraffin embedding. Coronal 6 micro-m sections from individual bones were sectioned and multiple sections from 4 mice per group used in subsequent procedures. Prior to staining sections were dewaxed and rehydrated.

1. **Toluidine blue staining**: sections were stained with toluidine blue (0.1% in 0.1M solution of acetate buffer [pH 5.6]), mounted with DPX mounting medium and imaged using a DM4000B upright microscope and DC500 colour camera both controlled through Leica Application Suite software version 2.8.1 (Leica Microsystems, Milton Keynes, UK). Growth plate zones were identified based on cell morphology and organisation, measured and expressed as a proportion of the total growth plate width using ImageJ. All growth plate zones were identified and measured by the same observer, and the growth plate images were temporarily assigned a random ID number unrelated to treatment group during analysis to minimise bias.
2. **Alcian Blue Haematoxylin and Orange G/Eosin staining**: sections were incubated with 1% acid-alcohol (1% hydrochloric acid made in 70% ethyl alcohol), stained with Alcian blue/haematoxylin (0.5% haematoxylin, 5% aluminium ammonium sulfate, 0.05% sodium iodate, 0.5% Alcian blue, 50% glycerol, 0.02% glacial acetic acid), washed in distilled H_2_O and differentiated in acid-alcohol. Sections were then stained with eosin–orange G (1.2% eosin in 90% alcohol plus 1% phloxine B and 2% orange G) for 1 min 30 seconds, dehydrated. Sections were mounted with DPX mounting medium and imaged using a DM4000B upright microscope and DC500 colour camera both controlled through Leica Application Suite software version 2.8.1 (Leica Microsystems, Milton Keynes, UK). Cartilage stains pale blue, bone orange-red, muscle red and bone marrow dark blue. These sections were analysed using Osteoid Histo [46] to provide trabecular thickness, total area, bone area and bone perimeter
3. **Tartrate resistant acid phosphatase (TRAP) staining**: paraffin-embedded histological sections were stained for TRAP activity using the standard naphthol AS-BI phosphate post coupling method. The slides were incubated for 1 hour at 37°C in 0.92% sodium acetate buffer, pH 5.0, containing 0.01% naphthol AS-BI phosphate and 1.14% L-(+)Tartaric acid. Then, the sections were incubated in the same buffer containing 0.1% pararosaniline chloride for 20 min, followed by washing in distilled water. The sections were counterstained with 0.5% methyl green (pH 4.2, nuclei blue) for 5 minutes, dehydrated and mounted with DPX mounting medium. Sections were imaged using a DM4000B upright microscope and DC500 colour camera both controlled through Leica Application Suite software version 2.8.1 (Leica Microsystems, Milton Keynes, UK). TRAP-positive osteoclasts were quantified using TRAP Histo [46] which identifies trabecular bone and osteoclasts by colour thresholding in combination with object filtering tools below the growth plate excluding cortical bone in a blinded fashion. Osteoclast numbers were expressed as N.Oc/BPm and Oc.S/BS according to accepted histomorphometric standard. Endosteal osteoblasts and number of osteocytes (lacunar occupancy) measurements were made in ImageJ cell counter plugin using x40 images taken from same region of cortical bone.

### Statistical analysis

Trabecular and skull bone data were analysed and box-plots generated using GraphPad Prism 6 (GraphPad Software, Inc., San Diego, CA). For cortical bone, graphs were developed using the R programming language “R”, version 3.1.3 (R Foundation for Statistical Computing, Vienna, Austria; http://www.r-project.org). Normality and homogeneity of variance of all the data were checked using the Shapiro-Wilk and the Bartlett’s test in the R 3.1.3 respectively. Two-sample t-test was used to compare means between KO and WT_B6_, and between Tg and WT_F1_. Kruskal-Wallis test was employed if either the normality or the homogeneity of variance assumptions were violated (p ≥ 0.05). Data are presented as mean ±SEM and were considered statistically significant when p ≤ 0.05.

## Results

### Deficiency and also transgenic overexpression of *Timp-3* generate defects in trabecular bone

**Fig 1:**
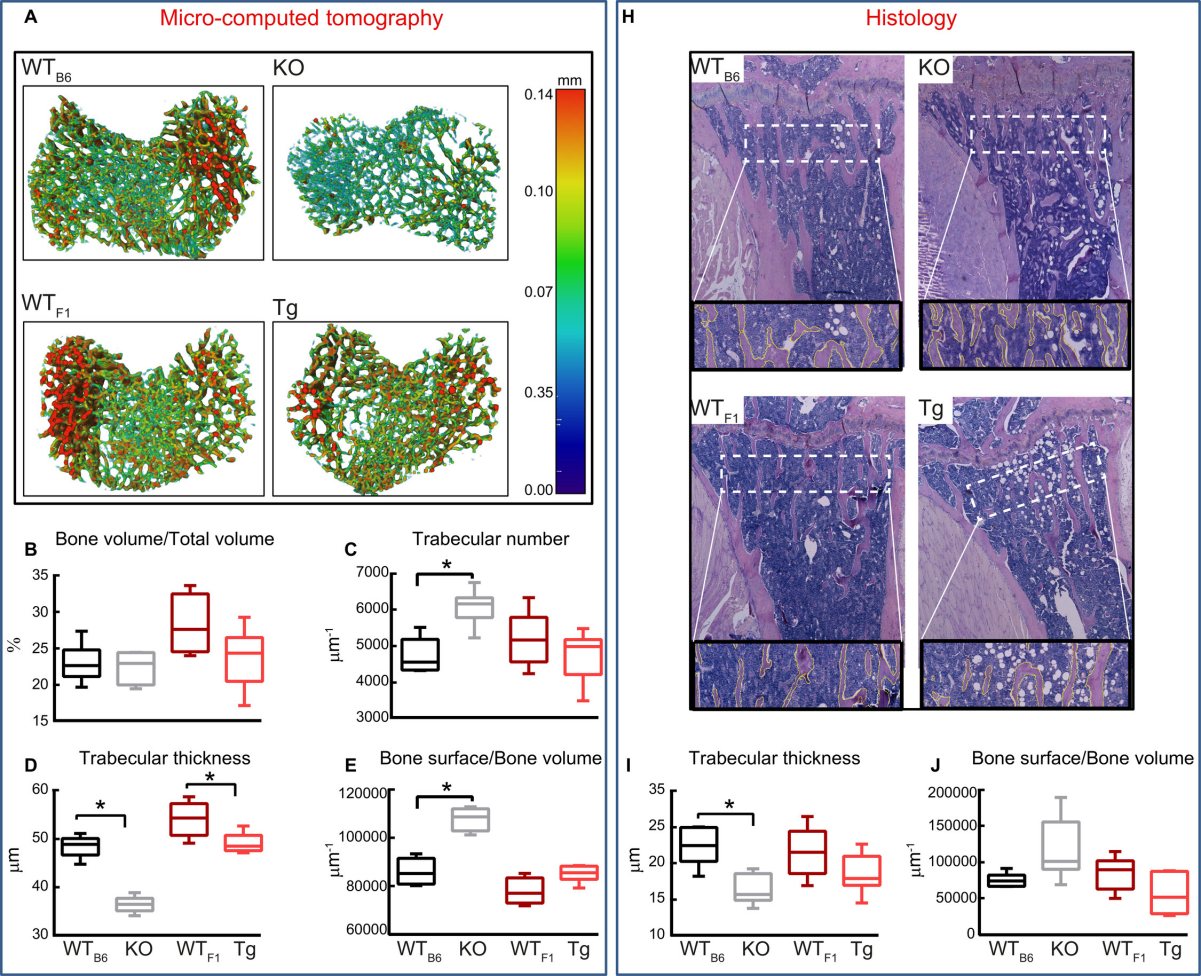
Deficiency and also transgenic overexpression of *Timp-3* generate defects in trabecular bone. Trabecular bone phenotype of WT_B6_ (black), *Timp-3* KO (grey), WT_F1_ (dark red), *Timp-3* Tg (light red) tibia at 8 weeks of age. (*A*) Representative 3D Micro-CT thickness colour-coded images of tibial trabecular bone. *Ex vivo* high-resolution analyses of distal proximal metaphysical tibia to determine (*B*) trabecular bone volume/total volume, (*C*) trabecular number, (*D*) trabecular thickness and (*G*) bone surface/bone volume. (*H*) Representative Alcian blue haematoxylin and orange G/eosin stained sections with outline of trabeculae showing the region of interest used for measurements. These sections were analysed using Osteoid Histo to provide (*I)* trabecular thickness and *(J)* trabecular bone surface/bone volume. Box-plots represent means ± SEM. Group sizes for Micro-CT (B-G) were *n*  =  5 for WT littermates and n = 6 for *Timp-3* KO and Tg mice. Group sizes for histological data (I-L) were *n*  =  4 for all groups. Two-sample t-test was used to compare means between KO and WT_B6_, and between Tg and WT_F1_. Normality of variance assumption was violated for total area of KO group (*E*) and homogeneity for trabecular thickness of WT *vs*. KO group (*I*) (p > 0.05), thus, for these groups Kruskal-Wallis test was performed. Statistical comparisons: * denotes *p* ≤ 0.05.

Herein, we used Micro-CT to perform a detailed analysis of bone morphology in 8 week old male *Timp-3* KO (and WT_B6_) and *Timp-3* homozygote for the transgene (Tg/Tg and WT_F1_ mice; Fig 1A). Our data show that *Timp-3* deficiency resulted in significantly shorter tibia compared with WT_B6_ (p ≤ 0.05, Table 1), however, no significant differences were observed between Tg and WT_F1_ mice. Furthermore, we found that both *Timp-3* deficiency and transgenic expression resulted in lower bone mineral density (BMD) in both trabecular and cortical compartments compared with their respective WTs; these changes only reached levels of statistical significance in KO mice compared with WT_B6_ control (p ≤ 0.01, Table 1). In addition, Micro-CT based comparison of the tibial trabecular bone revealed significantly lower total volume (TV, p ≤ 0.05, Table 1) and bone volume (BV) in *Timp-3* KO compared with WT littermates (Table 1 p < 0.05), but no significant differences in bone volume fraction (BV/TV) (Fig 1B). In contrast, no significant difference in TV, BV and BV/TV were observed between *Timp-3* Tg and WT_F1_ mice (Table 1 and Fig 1B). Trabecular number and bone surface/bone volume (BS/BV) were significantly higher in *Timp-3* KO than in WT_B6_ mice (Fig 1C and 1E; p ≤ 0.05), however, no such differences were found in *Timp-3* Tg mice. Trabecular thickness was significantly lower in both *Timp-3* KO and Tg mice compared with respective WT mice (Fig 1A and 1D; p ≤ 0.05).

**Table 1:** Mean value of morphometric parameters from the 2D and 3D analysis representing trabecular and cortical mass and architecture of WT_B6_, *Timp-3* KO, WT_F1_ and *Timp-3* Tg mice at 8 weeks of age.

Morphometric index	WT_B6_	KO	P value	WT_F1_	Tg	P value
	n = 5	n = 6	WT_B6_ vs KO	n = 5	n = 6	WT_F1_ vs Tg
Tibial length (mm)	18.11 ± 0.02	16.45 ± 0.06	<0.001	17.33 ± 0.05	17.32 ± 0.02	NS
**Trabecular parameters**						
Trabecular BMD g/cm^3^	0.304 ± 0.01	0.263 ± 0.01	<0.05	0.409 ± 0.02	0.350 ± 0.01	NS
Total volume (micro-m^3^) x 10^9^	2.34 ± 0.12	1.74 ± 0.09	<0.01	1.99 ± 0.09	2.00 ± 0.07	NS
Bone volume (micro-m^3^) x 10^8^	5.45 ± 0.50	3.88 ± 0.18	<0.05	5.63 ± 0.59	4.78 ± 0.42	NS
Trabecular separation (mm)	0.14 ± 0.00	0.12 ± 0.00	<0.05	0.14 ± 0.01	0.15 ± 0.01	NS
**Cortical parameters**						
Cortical BMD g/cm^3^	0.505 ± 0.04	0.334 ± 0.03	<0.01	0.592 ± 0.03	0.554 ± 0.05	NS

Group sizes for were *n*  =  5 for WT littermates and n = 6 for *Timp-3* KO and Tg mice, except for n = 4 for all groups for BMD. Two-sample t-test was used to compare means between KO and WT_B6_, and between Tg and WT_F1_. Normality or the homogeneity of variance assumption were not violated (p ≥ 0.05). Data are mean ± SEM.

We also found that analysis of trabecular bone from Alcian blue/ haematoxylin and orange G/eosin stained sections (Fig 1H) resulted in similar differences between KO and Tg and their respective WT mice (Fig 1I and 1J) which allowed confirmation of our findings obtained from micro-computed tomography. Together these data reveal that *Timp-3* deficiency produces smaller metaphyseal trabecular area, lower BV and more numerous, thinner trabeculae. *Timp-3* transgenic overexpression does not produce opposite changes, as it also compromises trabecular structure by reducing their thickness.

### Deficiency and also transgenic overexpression of *Timp-3* do not lead to significant alteration in growth plate

**Fig 2:**
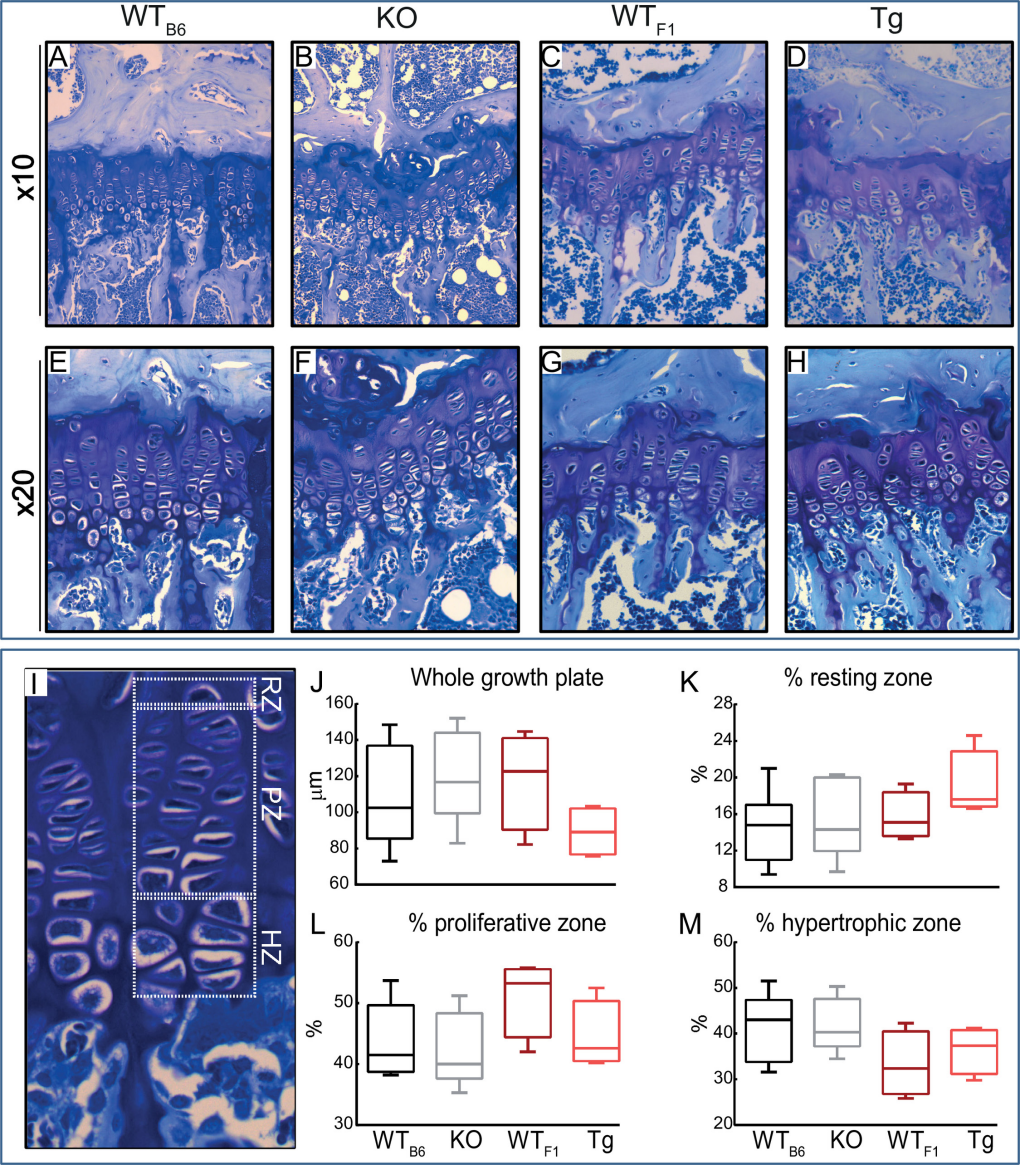
*Timp-3* deficiency and transgenic chondrocyte-specific overexpression do not alter thickness of different zones in growth plate. Representative images of toluidine blue stained sections from WT_B6_ (A and E), KO (B and F), WT_F1_ (C and G) and Tg (D and H) mice showing overall structure of growth plate measured from resting zone to primary spongiosa. (I) Different zones of growth plate were marked and measured. No significant differences in size overall size of growth plate (J), percent proliferative zone (K), percent hypertrophic zone (L) and percent resting zone (M) between KO and their WT controls as well as Tg and their respective WT were observed. Two-sample t-test was used to compare means between KO and WT_B6_, and between Tg and WT_F1_. Normality and homogeneity of variance assumptions were not violated in any experimental group (p ≥ 0.05). Group sizes were *n*  =  4. Data are mean ± SEM.

In sections of long bones from hind limbs (Fig 2A–2M), the length of the growth plates in WT_B6_ (Fig 2A and 2E), KO (Fig 2B and 2F), WT_F1_ (Fig 2C and 2G) and Tg (Fig 2D and 2H) mice showing overall structure of growth plate (measured from resting zone to primary spongiosa) in 8 week old mice were not significantly altered. The length of the total growth plate in proximal tibias was not altered in KO and Tg mice compared with their WT mice. Furthermore, proliferative (K), hypertrophic (L) and resting zones (M) expressed as a percentage of total growth plate were not significantly different.

### *Timp-3* deficiency and transgenic chondrocyte-specific overexpression produce gross changes in tibial anatomy

**Fig 3:**
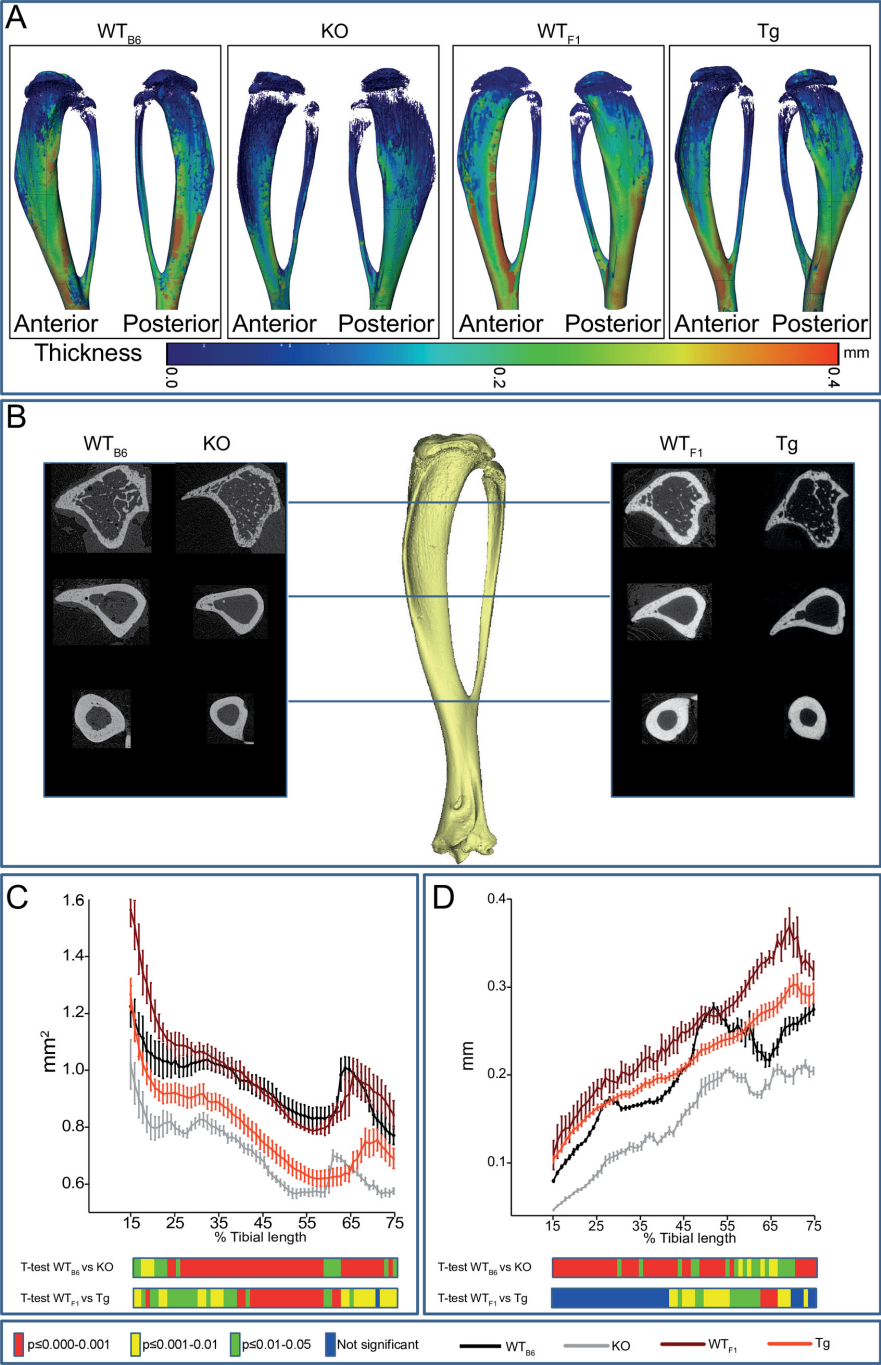
*Timp-3* deficiency and transgenic chondrocyte-specific overexpression produce gross changes in cortical bone. *(A)* Representative 3D Micro-CT colour-coded images of tibial cortical bone thickness. *(B)* Comparable cross-sectional reconstructed 2D images along the length of the tibia. *(C)* Bone cross sectional area (CSA) and (D) mean cortical thickness of WT_B6_ (black), *Timp-3* KO (grey), WT_F1_ (dark red) and *Timp-3* Tg (light red) tibia at 8 weeks of age. Whole bone analyses of cortical bone between 15–75% of total tibial length, excluding proximal and distal metaphyseal bone. Line graphs represent means ± SEM. Group sizes were *n*  =  5 for WT littermates and n = 6 for KO and Tg mice. Two-sample t-test was used to compare means between KO and WT_B6_, and between Tg and WT_F1_. Graphical heat map summarises statistical differences at specific matched locations along the tibial length, representation of the overall effect of genotype and post-hoc analysis are also shown. Red p≤0.000–0.001, yellow p≤0.001–0.01, green p≤0.01–0.05 and blue p ≥ 0.05.

To determine whether *Timp-3* KO and Tg mice also show modified cortical tibial bone architecture we undertook whole-bone analysis (Fig 3A). We found that genotype (*Timp-3* deficiency or overexpression) was a significant determinant of bone cross-sectional area (CSA; Fig 3C), producing lower bone CSA in *Timp-3* KO and Tg mice compared with WT_B6_ and WT_F1_ mice, respectively along the entire tibia length (Fig 3C). We found that genotype also contributed significantly to cortical thickness (Fig 3D), with *Timp-3* KO mice exhibiting lower thickness than WT_B6_ mice. Reduction in cortical thickness, albeit less marked, was also observed in *Timp-3* Tg mice particularly towards the distal tibia (Fig 3C). We also observed differing patterns of thickness along the length of the tibial shaft in WT_B6_ and WT_F1_ mice.

**Fig 4:**
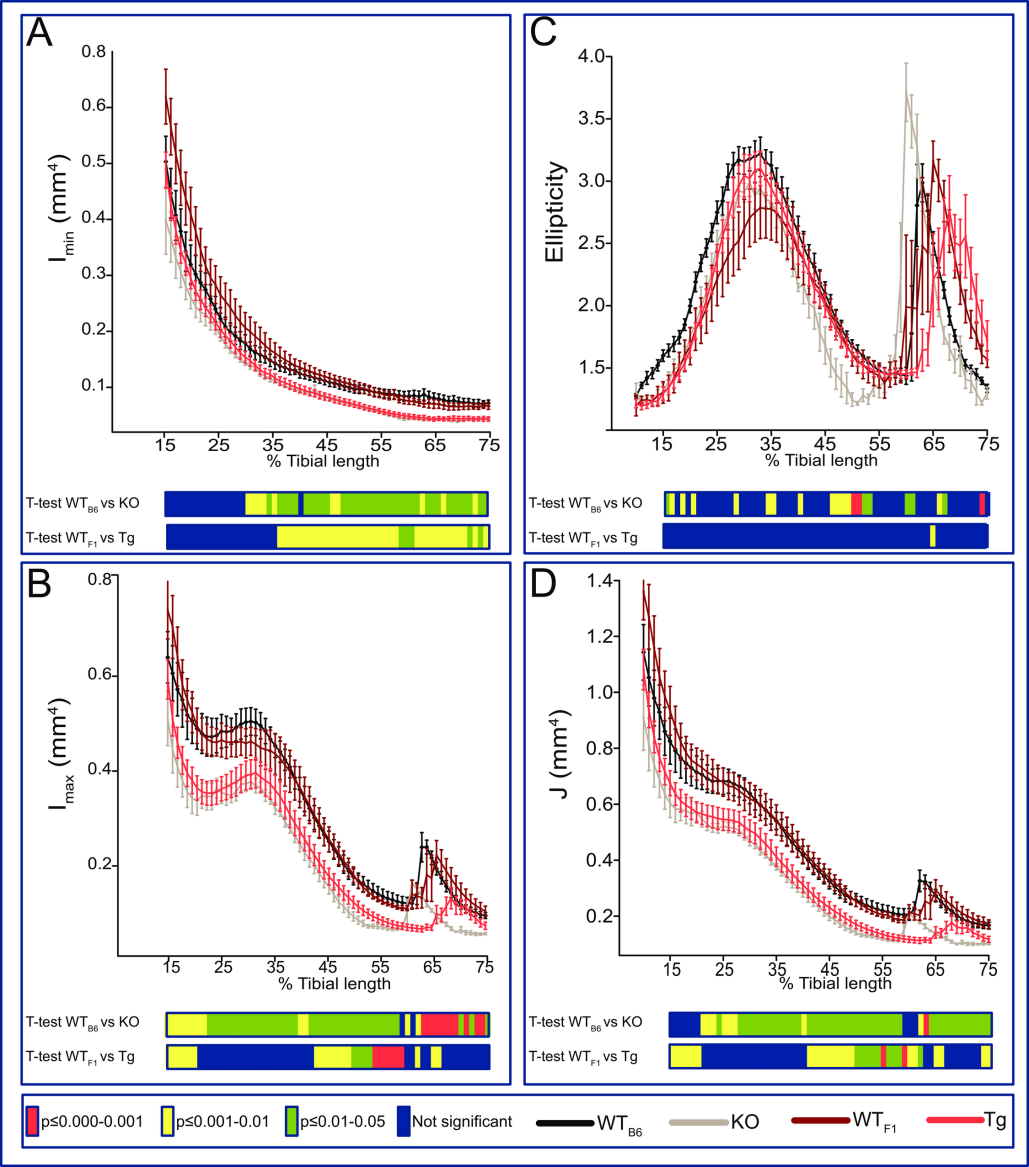
*Timp-3* deficiency and transgenic chondrocyte-specific overexpression produce gross changes in tibial geometry. (A) Minimum and (B) maximum second moments of area (I_min_ and I_max_ respectively), (C) ellipticity and J (D; resistance to torsion) of WT_B6_ (black), *Timp-3* KO (grey), WT_F1_ (dark red) and *Timp-3* Tg (light red) tibia at 8 weeks of age. Whole bone analyses of cortical bone between 15–75% of total tibial length, excluding proximal and distal metaphyseal bone. Two-sample t-test was used to compare means between KO and WT_B6_, and between Tg and WT_F1_. Line graphs represent means ± SEM. Group sizes were *n*  =  5 for WT littermates and n = 6 for KO and Tg mice. Graphical heat map summarises statistical differences at specific matched locations along the tibial length, representative of overall effect of genotype and post-hoc analysis are also shown. Red p≤0.000–0.001, yellow p≤0.001–0.01, green p≤0.01–0.05 and blue p ≥ 0.05.

To provide an estimate of tibial resistance to bending forces, we calculated second moment of area around minor (I_min_) and major axes (I_max_). This showed that the overall effect of genotype on I_min_ was most pronounced in *Timp-3* KO distal to the mid-shaft (Fig 4A) with a lack of marked proximal tibia modification (Fig 4A). Transgenic *Timp-3* overexpression also, surprisingly, produced lower I_min_ along the tibial shaft (Fig 4A). I_max_ was lower along almost the entire tibia of *Timp-3* KO and in Tg mice (Fig 4B). Tibial ellipticity was also modified by genotype (Fig 4C), with several locations along the tibia showing greater ellipticity in *Timp-3* KO, but not *Timp-3* Tg mice compared to WT mice (Fig 4C). Predicted tibial resistance to torsion is lower in both KO and surprisingly in Tg mice than in their corresponding WT mice (Fig 4D). Our data indicate that both *Timp-3* deficiency and chondrocyte-driven *Timp-3* overexpression produce deficits in cortical bone mass, however only the former also produces statically significant additional changes in cortical bone shape; some changes in shape might occur in *Tg* mice but these are not significant. Consequences of these modifications are decreases in predicted bone strength in both *Timp-3* KO and *Timp-3* Tg mice, despite the targeting of *Timp-3* overexpression via a chondrocyte-specific promoter.

### *Timp-3* deficiency results in increased osteoclast number and TRAP staining

**Fig 5:**
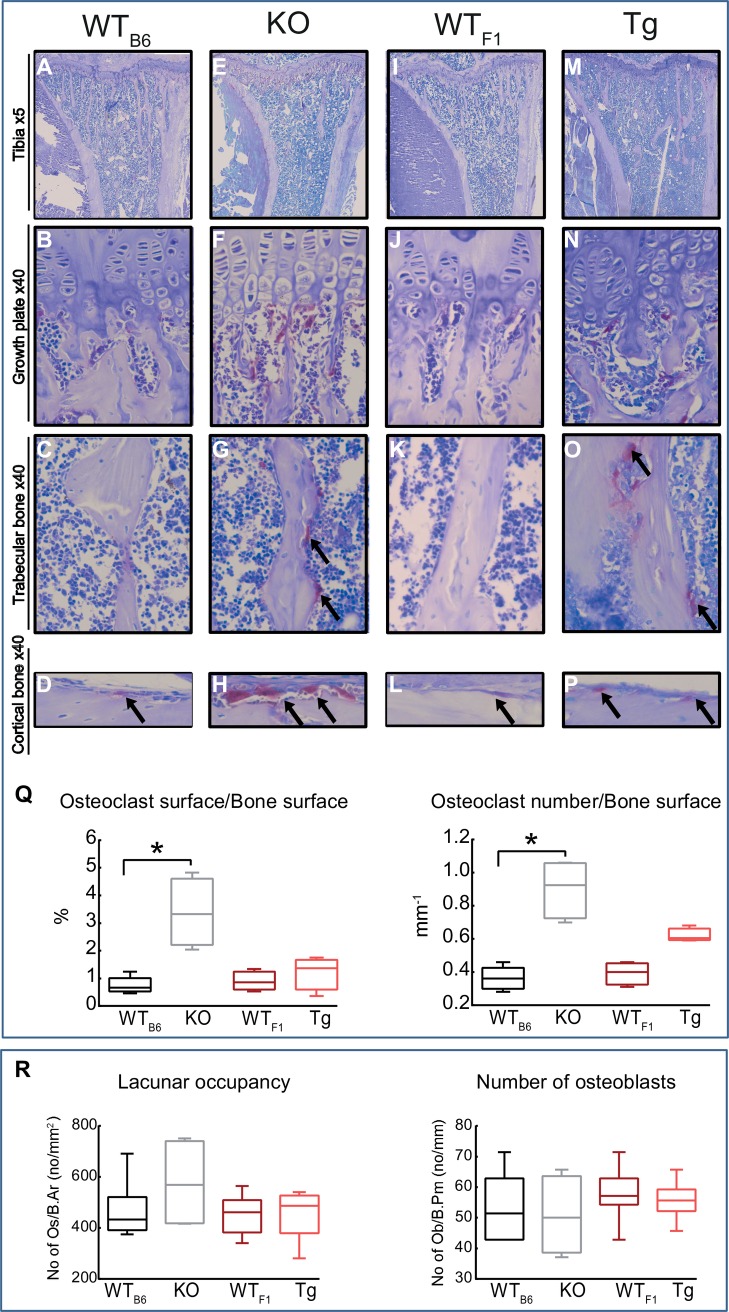
*Timp-3* deficiency and transgenic chondrocyte-specific overexpression increase trabecular TRAP staining. Representative images of TRAP stained sections from WT_B6_ (A-D), KO (E-H), WT_F1_ (I-L) and Tg (M-P) mice demonstrating TRAP activity in growth plate, trabecular and cortical bone. Staining show that TRAP activity is significantly higher in KO and Tg sections compared with their WT groups. (Q) TRAP activity was quantified to produce osteoclast surface/bone surface and osteoclast number/bone surface. Two-sample t-test was used to compare means between KO and WT_B6_, and between Tg and WT_F1_. Normality of variance assumption were not violated for any experimental group. Homogeneity of variance osteoclast surface/bone surface between KO and WT_B6_ was violated (p >0.05) and thus for these groups Kruskal-Wallis test was performed. Group sizes were *n*  =  4. Data are mean ± SEM. Statistical comparisons: * denotes *p* ≤ 0.05.

To determine whether *Timp-3* deficiency and/or transgenic overexpression alter bone resorption, we performed TRAP staining in tibial sections and measured bone resorption indices. Our data show that *Timp-3* deficiency produces significantly higher number of osteoclasts in both trabecular (p ≤ 0.01; Fig 5Q) and cortical compartments (not quantified) compared with WT_B6_ mice (Fig 5A–5H) and that, in contrast such increases do not reach levels of statistical significance in Tg mice compared with WT_F1_ controls (Fig 5I–5Q). Furthermore, *Timp-3* deficiency leads to significant increases in the ratio of osteoclast surface to bone surface as well as osteoclast number to bone surface (p ≤ 0.01; Fig 5Q), however, changes due to transgenic expression were not statistically significant. We also measured the number of endosteal osteoblasts expressed as a ratio to bone perimeter and lacunar occupancy expressed as a ratio of bone area and found neither *Timp-3* deficiency nor transgenic expression significantly alter these indices (p ≤ 0.01; Fig 5R).

### *Timp-3* deficiency and overexpression produce architectural changes in skull

**Fig 6:**
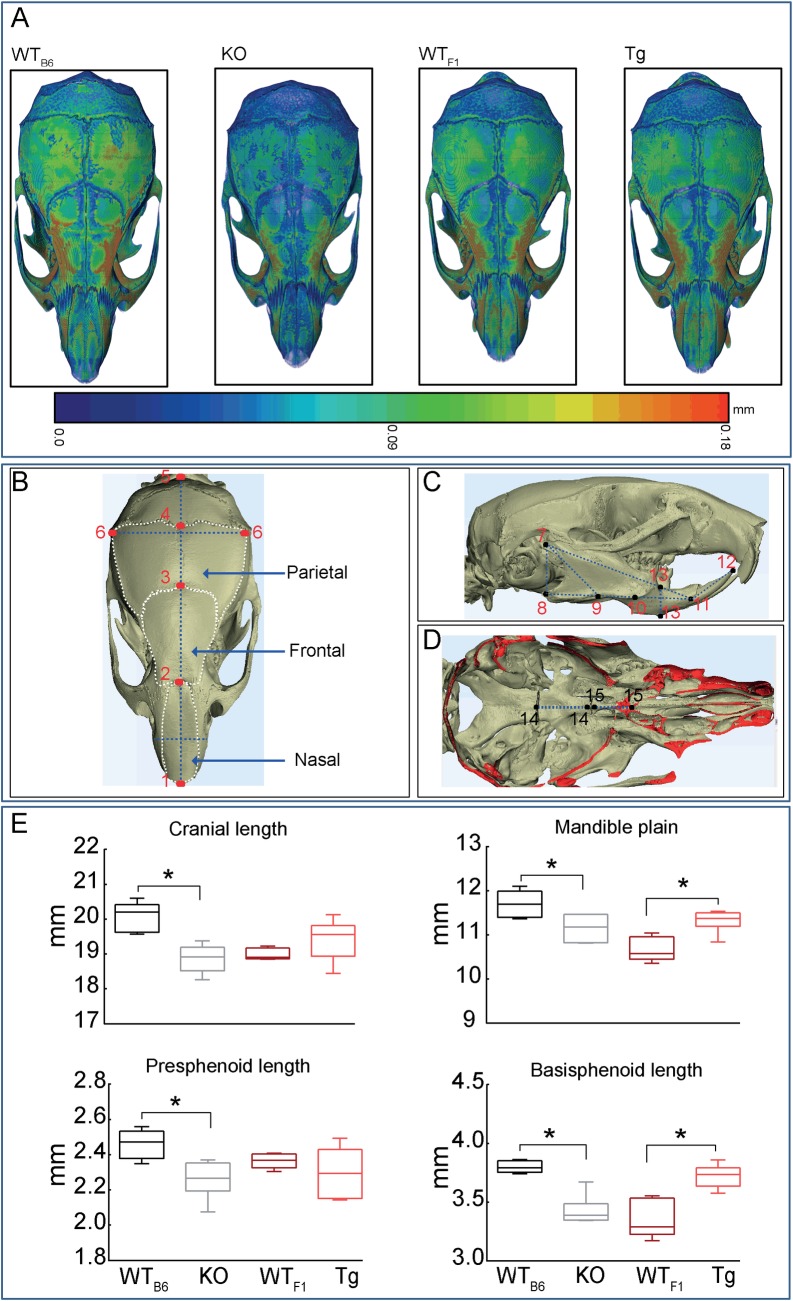
*Timp-3* deficiency and overexpression produce architectural changes in the skull. Craniometric measurements of WT_B6_, *Timp-3* KO, WT_F1_ and *Timp-3* Tg skull at 8 weeks of age. (*A*) Representative 3D Micro-CT colour-coded images of skull thickness. (*B*) Schematic of the structures of the mouse skull depicting landmarks used to obtain measurements in the cranial; 1–2: nasal length, 2–3: frontal length, 3–4: parietal length, 1–5: cranial length, 6–6: bitemporal distance, *(C)* 7–8: posterior mandible height, 7–9: condilar axis, 7–11: effective mandible length, 8–11: mandible plain, 9–10: mandible axis, 11–12: inferior incisor axis, 13–13: *(D)* anterior mandible height, 14–14: basisphenoid length, and 15–15: presphenoid length in which the ‘shell’ regions of the bone that correspond to plane of sectioning are coloured in red. (*E*) Cranial length, mandible plain, presphenoid and basisphenoid length. Box-plots represent means ± SEM. Group sizes were *n*  =  5. Statistical comparisons: * denotes *p* ≤ 0.05.

To explore whether these skeletal effects of *Timp-3* deficiency or overexpression were similar in bones with divergent origins, we compared cranial anatomy of *Timp-3* KO and Tg mice to controls (WT_B6_ and WT_F1_ respectively). Eighteen measurements were made to evaluate bones formed by intramembranous ossification from neural crest cell (nasal, frontal and mandible) or mesoderm origins (parietal), and those formed by endochondral ossification with either neural crest (presphenoid) or mesodermal origins (basisphenoid) [47–50]. Landmarks used to obtain measurements are depicted in Fig 6B–6D. Our data show that *Timp-3* deficiency reduced skull thickness as demonstrated by colour-coded image (Fig 6A). Moreover, *Timp-3* deficiency produces shorter overall cranial length (p ≤ 0.01; Fig 6E) without significant effect on the length or area of any of the contributory, individual intramembranously-formed skull bones with neural crest (nasal and frontal) or mesoderm (parietal) origins. No such significant modification in overall cranial length was apparent in the *Timp-3* Tg mice (Fig 6E).

*Timp-3* KO mice also showed significantly smaller mandible plain and, in accord with our hypothesis concerning opposite effects of Tg overexpression, this skull region showed a corresponding expansion in *Timp-3* Tg mice (Fig 6E; p ≤ 0.05 and p ≤ 0.01 respectively). *Timp-3* deficiency also affected other mandibular landmarks (condylar and inferior incisor axis and anterior mandible height; Table 2; p ≤ 0.05); *Timp-3* Tg mice showed no significant effects on the mandible. Effects of *Timp-3* deficiency were similar in the endochondrally-derived basisphenoid bone from mesoderm origins (Fig 6E; p < 0.001). The presphenoid bone (endochondral–neural crest) was however only affected in *Timp-3* KO mice (Fig 6E; p < 0.05). Together, these data show that *Timp-3* deficiency affects skull bones formed by both endochondral and intramembranous mechanisms whether they are derived from neural crest or mesodermal origins. As predicted, and in apparent contrast to its effects in long bones, *Timp-3* Tg overexpression via a chondrocyte-specific promoter produces far more restricted but, only ever, opposite effects.

**Table 2:** Craniometric measurements representing skull parameters of WT_B6_, KO, WT_F1_ and Tg mice at 8 weeks of age.

Craniometric measurements	WT_B6_	KO	P value	WT_F1_	Tg	P value
	n = 5	n = 5	WT_B6_ vs KO	n = 5	n = 5	WT_F1_ vs Tg
**Intramembranous (neural crest)**						
Internasal distance (mm)	3.32 ± 0.02	3.42 ± 0.04	NS	3.29 ± 0.07	3.49 ± 0.04	NS
Nasal length (mm)	5.38 ± 0.08	5.19 ± 0.16	NS	5.65 ± 0.21	5.42 ± 0.15	NS
Nasal area (mm^2^)	9.35 ± 0.33	9.71 ± 0.50	NS	10.67 ± 0.64	9.77 ± 0.52	NS
Frontal length (mm)	7.09 ± 0.20	7.01 ± 0.13	NS	7.18 ± 0.09	6.75 ± 0.16	NS
Frontal area (mm^2^)	25.62 ± 1.42	27.86 ± 1.25	NS	28.70 ± 0.92	25.72 ± 1.11	NS
**Intramembranous (mesoderm)**						
Bitemporal distance (mm)	8.78 ± 0.05	8.49 ± 0.11	NS	8.54 ± 0.15	8.60 ± 0.09	NS
Parietal length (mm)	4.07 ± 0.11	4.09 ± 0.09	NS	3.84 ± 0.06	3.81 ± 0.08	NS
Parietal area (mm^2^)	39.36 ± 2.10	36.93 ± 1.56	NS	34.93 ± 0.66	38.09 ± 1.30	NS
**Mandible, intramembranous (neural crest)**						
Effective mandible length (mm)	11.90 ± 0.07	11.78 ± 0.07	NS	11.41 ± 0.22	11.69 ± 0.14	NS
Mandible axis (mm)	2.64 ± 0.10	2.76 ± 0.06	NS	2.15 ± 0.03	2.34 ± 0.79	NS
Condilar axis	5.76 ± 0.04	6.31 ± 0.18	<0.05	5.70 ± 0.08	5.84 ± 0.05	NS
Inferior incisor axis (mm)	4.34 ± 0.12	3.72 ± 0.11	<0.05	3.74 ± 0.02	3.73 ± 0.08	NS
Anterior mandible height (mm)	2.39 ± 0.01	2.59 ± 0.03	<0.05	2.37 ± 0.45	2.29 ± 0.06	NS
Posterior mandible height (mm)	3.98 ± 0.16	3.58 ± 0.11	NS	3.23 ± 0.07	3.46 ± 0.11	NS

Two-sample t-test was used to compare means between KO and WT_B6_, and between Tg and WT_F1_. Normality and homogeneity of variance assumptions were not violated in any experimental group (p ≥ 0.05). Data represent means ± SEM. Statistical comparisons: *p* ≤ 0.05.

## Discussion

MMPs are important regulators of skeletal homeostasis and thus understanding how their endogenous regulators affect bone mass and architecture will provide novel insights. We have used Micro-CT to assess bone mass and architecture as well as histological and histochemical evaluation to characterise the bone phenotype of *Timp-3* KO mice, complemented by similar examination in mice harbouring a *Timp-3* transgene driven via a Col-2a-driven promoter to specifically target overexpression to chondrocytes. We have used these mice to address three questions: i) how does *Timp-3* deficiency modify tibial bone mass and architecture, ii) to what extent does cartilage-specific *Timp-3* overexpression produce opposite effects, and iii) can examination of particular skull bones, with well-established differences in developmental origins and distinct mechanisms of formation, help to identify the functions of *Timp-3* in bone.

Our detailed analyses reveal that *Timp-3* deficiency and, less so, Tg overexpression generate defects in tibial trabecular structure and compromise cortical bone along the entire shaft. These observed alterations in cortical mass are predicted to significantly compromise tibial load-bearing resistance to torsion in both genotypes. Based on previous studies using MMP knockout mice, we predicted that *Timp-3* deficiency would compromise bone mass and structure, and perhaps bone mineral density. For example, MMP-13 has been shown to act as a negative regulator of bone formation and promoter of bone resorption [28, 51, 52]. In the skeleton, MMP-13 is expressed in hypertrophic chondrocytes and in osteoblasts during development, and in remodelling bone postnatally [28, 53] and is thus a likely target for TIMP-3. MMP-13 KO mice exhibit increases in trabecular volume/total bone volume, trabecular number and trabecular thickness, and decreases in trabecular separation. Our findings showing opposite architectural changes in *Timp-3* KO mice are therefore consistent with the proposed role of *Timp-3* as a suppressor of MMP-13. We speculate that the compromised bone in *Timp-3* deficient mice is the result of decreased bone formation and increased resorption and does not involve any intermediary cartilage-mediated contributions [3]. Alternatively, *Timp-3* KO may accelerate osteoblast proliferation and differentiation, thereby depleting the available stem cell pool and compromised bone remodelling [29, 54]. Our examination by TRAP staining shows that *Timp-3* KO mice show marked elevation of osteoclast numbers and CT reveals that this is accompanied by decreases in bone mineral density, that appear to provide some mechanistic basis for the observed decreases in bone mass in these mice. These data indicate that *Timp-3* contributes to the attainment of functionally-appropriate tibial bone mass and architecture that is likely achieved via regulation of osteoclast numbers/function.

Failure to identify similar marked increases in osteoclast numbers or decreases in bone mineral density in Tg mice pinpoint a need to define the mechanisms by which decreased bone mass is achieved. Bone mass and architectural changes observed in Tg mice were indeed, in contrast, somewhat unexpected. We found there to be deleterious effects on long bones, derived endochondrally, as well as some changes in bones of the skull derived via intramembranous ossification. Our use of mice harbouring a Col2a1 promoter-driven transgene was selected to target *Timp-3* overexpression to cartilage and, thus, we had predicted that the effects on bone mass and architecture may be restricted to only bones formed via endochondral ossification, and that these effects would be opposite to those observed in *Timp-3* KO mice. This impact would likely be via incorporation of TIMP-3 into the cartilage template for bone formation at greater levels than in WT mice, with downstream indirect effects on osteoblast and osteoclast function. Our findings are also in disagreement with data by Shen *et al*., (2010) in which an increased trabecular bone volume observed in Tg mice in which hematopoietic stem cells were retrovirally transduced with human TIMP-3. It is possible that differences in the targeting of *Timp-3* in these two transgenic mouse models to either hematopoietic stem cells or chondrocytes may underlie the differences in phenotypes observed. We have used a collagen type II promoter, thus, deleterious phenotype observed in Tg mice may also indicate that collagen type II is expressed at some stages in bone cells and that its overexpression leads to a dose-dependent effect resulting in a compromised bone structure. This is supported by a number of previous [55–63] and more recent studies [32, 34, 64, 65] challenging current dogma, showing that hypertrophic chondrocytes can transdifferentiate into osteoblasts during endochondral bone formation. This may lead to continued expression of collagen type II and consequently *Timp-3*, resulting in inhibition of osteoblast differentiation [66]. Our data revealing no changes in endosteal osteoblast number or osteocyte lacunar occupancy in either *Timp-3* KO or Tg mouse tibial cortices suggest that any cells making such hypertrophic chondrocyte-to-osteoblast transition do not confer changes in osteoblast behaviour.

This possibility is, however, inconsistent with our findings in intramembranous bones of the skull where Tg show a clear phenotype. Examination of different skull bones from different developmental origins revealed that *Timp-3* affects bones from both endochondral and intramembranous derived processes [67]. We found that *Timp-3* deficiency leads to reduction in length in 7 structures developed both from endochondral and intramembranous ossification, whereas, *Timp-3* overexpression leads to increased length of two structures developed from both endochondral and intramembranous ossification. These observations suggest a direct bone specific role for *Timp-3* in craniofacial development. Our data showing that the zonal organisation and size of the growth plate in both 8 week old *Timp-3* KO and Tg mice is not modified, suggest that their defective bone phenotypes do not result from any global effect on endochondral ossification. The exact mechanisms underpinning the effects of *Timp-3* deficiency or overexpression are yet to be fully resolved however our data clearly demonstrate that *Timp-3* regulates bone mass and architecture *in vivo* and suggests that TIMP-3 could be a target for modulation of bone mass and architecture.

## Supporting Information

S1 Fig Deficiency and also transgenic overexpression of *Timp-3* generate defects in trabecular bone. Morphometric parameters from the 2D and 3D analysis representing trabecular mass and architecture of WT_B6_, *Timp-3* KO, WT_F1_ and *Timp-3* Tg mice at 8 weeks of age.
(XLSX)

S2 Fig *Timp-3* deficiency and transgenic chondrocyte-specific overexpression do not alter thickness of different zones in growth plate. Data from toluidine blue stained sections from WT_B6_, KO, WT_F1_ and Tg mice representing overall structure of growth plate measured from resting zone to primary spongiosa.
(XLSX)

S3 Fig *Timp-3* deficiency and transgenic chondrocyte-specific overexpression produce gross changes in cortical bone. Whole bone analyses of cortical bone excluding proximal and distal metaphyseal bone showing bone cross sectional area (CSA) and mean cortical thickness of WT_B6_, *Timp-3* KO, WT_F1_ and *Timp-3* Tg tibia at 8 weeks of age.
(XLSX)

S4 Fig *Timp-3* deficiency and transgenic chondrocyte-specific overexpression produce gross changes in tibial geometry. Whole bone analyses of cortical bone excluding proximal and distal metaphyseal bone showing minimum and maximum second moments of area (I_min_ and I_max_ respectively), ellipticity and J (resistance to torsion) of WT_B6_, *Timp-3* KO, WT_F1_ and *Timp-3* Tg tibia at 8 weeks of age.
(XLSX)

S5 Fig *Timp-3* deficiency and transgenic chondrocyte-specific overexpression increase trabecular TRAP staining. Data obtained from TRAP stained sections from WT_B6_, KO, WT_F1_ and Tg mice demonstrating TRAP activity in trabecular bone.
(XLSX)

S6 Fig *Timp-3* deficiency and overexpression produce architectural changes in the skull. Craniometric measurements of WT_B6_, *Timp-3* KO, WT_F1_ and *Timp-3* Tg skull at 8 weeks of age.
(XLSX)

S1 Table Deficiency and also transgenic overexpression of *Timp-3* generate defects in trabecular bone. Morphometric parameters from the 2D and 3D analysis representing trabecular and cortical mass and architecture of WT_B6_, *Timp-3* KO, WT_F1_ and *Timp-3* Tg mice at 8 weeks of age.
(XLSX)

S2 Table *Timp-3* deficiency and overexpression produce architectural changes in the skull. Craniometric measurements of WT_B6_, *Timp-3* KO, WT_F1_ and *Timp-3* Tg skull at 8 weeks of age.
(XLSX)
